# Extracellular Vesicles from Human Cerebrospinal Fluid Are Effectively Separated by Sepharose CL-6B—Comparison of Four Gravity-Flow Size Exclusion Chromatography Methods

**DOI:** 10.3390/biomedicines10040785

**Published:** 2022-03-27

**Authors:** Vedrana Krušić Alić, Mladenka Malenica, Maša Biberić, Siniša Zrna, Lara Valenčić, Aleksandar Šuput, Lada Kalagac Fabris, Karmen Wechtersbach, Nika Kojc, Mario Kurtjak, Natalia Kučić, Kristina Grabušić

**Affiliations:** 1 Department of Physiology and Immunology, Faculty of Medicine, University of Rijeka, 51000 Rijeka, Croatia; vedrana.krusic@medri.uniri.hr (V.K.A.); natalia.kucic@medri.uniri.hr (N.K.); 2 Department of Medical Chemistry, Biochemistry and Clinical Chemistry, Faculty of Medicine, University of Rijeka, 51000 Rijeka, Croatia; mladenka.malenica@medri.uniri.hr; 3 General Hospital Pula, 52100 Pula, Croatia; masa.biberic@obpula.hr (M.B.); sinisa.zrna@obpula.hr (S.Z.); asuput@gmail.com (A.Š.); lada1966@gmail.com (L.K.F.); 4 Department of Anaesthesia and Intensive Care Medicine, Clinical Hospital Centre Rijeka, 51000 Rijeka, Croatia; lara.valencic@uniri.hr; 5 Department of Anaesthesia, Resuscitation and Intensive Care Medicine, Faculty of Medicine, University of Rijeka, 51000 Rijeka, Croatia; 6 Institute of Pathology, Faculty of Medicine, University of Ljubljana, 1000 Ljubljana, Slovenia; karmen.wechtersbachc@mf.uni-lj.si (K.W.); nika.kojc@mf.uni-lj.si (N.K.); 7 Advanced Materials Department, Jožef Stefan Institute, 1000 Ljubljana, Slovenia; mario.kurtjak@ijs.si

**Keywords:** extracellular vesicles, cerebrospinal fluid, traumatic brain injury, size exclusion chromatography, CD9 protein, CD81 protein, apolipoproteins

## Abstract

Extracellular vesicles (EVs) are a versatile group of cell-secreted membranous nanoparticles present in body fluids. They have an exceptional diagnostic potential due to their molecular content matching the originating cells and accessibility from body fluids. However, methods for EV isolation are still in development, with size exclusion chromatography (SEC) emerging as a preferred method. Here we compared four types of SEC to isolate EVs from the CSF of patients with severe traumatic brain injury. A pool of nine CSF samples was separated by SEC columns packed with Sepharose CL-6B, Sephacryl S-400 or Superose 6PG and a ready-to-use qEV10/70 nm column. A total of 46 fractions were collected and analysed by slot-blot followed by Ponceau staining. Immunodetection was performed for albumin, EV markers CD9, CD81, and lipoprotein markers ApoE and ApoAI. The size and concentration of nanoparticles in fractions were determined by tunable resistive pulse sensing and EVs were visualised by transmission electron microscopy. We show that all four SEC techniques enabled separation of CSF into nanoparticle- and free protein-enriched fractions. Sepharose CL-6B resulted in a significantly higher number of separated EVs while lipoproteins were eluted together with free proteins. Our data indicate that Sepharose CL-6B is suitable for isolation of EVs from CSF and their separation from lipoproteins.

## 1. Introduction

Extracellular vesicles (EVs) are cell-originated nanoparticles ranging in size from roughly 30 to 1000 nm and containing a hydrophilic inner core [1]. EVs can incorporate a diverse repertoire of proteins, RNA, and lipids, which may lead to varied biological activity in the recipient cell. EV characterisation has been supported by the advancements of inclusive databases (e.g., Vesiclepedia, ExoCarta, EVpedia) that amass EV findings from abundant studies with the aim of finding distinguishing molecular signatures to specific cell/tissue types [2,3,4]. Consequently, certain proteins, including classic EV markers, have been found to be present and may be used as EV markers in accordance with the minimum guidelines set by the International Society of Extracellular Vesicles (ISEV) [5]. An array of EV markers, such as the tetraspanin proteins (CD9, CD63, and CD81), flotillin-1/-2, ESCRT-related (Alix and TSG101), RABs, SNAREs, and others, have been reported in different disease models [6,7,8,9,10,11,12]. The EV surface is a lipid bilayer deriving either from: (1) the cell surface if EVs are released by budding from the plasma membrane; or (2) the cell interior if EVs are secreted by exocytosis after a multivesicular body has formed inside the cell [13]. While different cellular pathways might be used for EV secretion, the current view is that EV secretion is a common cellular process and that EVs can be secreted by cells of all types within physiological and pathophysiological conditions [14,15]. Furthermore, EVs are present in body fluids, which makes them a great source of biomarkers for molecular changes even in organs not readily accessible for biopsy, such as the brain [5].

Despite the remarkable progress achieved in EV research during the last decade, some technical issues still need to be resolved, particularly regarding the isolation of EVs from body fluids. Such isolation is challenging due to the inherent features of EVs, such as diversity in size, density, and molecular content on the surface, but also due to the characteristics of the body fluids, which differ in viscosity, pH, and in quantity and types of EVs, proteins, lipoproteins, and other nanoparticles. Depending on the isolation method, proteins and non-EV nanoparticles can co-isolate with EVs and thus impair the purity of the EV isolate. Consequently, diverse principles are applied to isolate EVs, focusing on EV size, density, and/or surface molecules. To date, the most commonly used method for isolating EVs is ultracentrifugation, followed by ultrafiltration and polymer-based precipitation, which have been extensively described in several reviews [16,17,18,19]. Although these methods enabled the EV field to develop and become very productive, they can result in low yield, aggregated, and deformed EVs mixed with contaminants such as free proteins and lipoproteins [20,21,22].

Size exclusion chromatography (SEC) is currently less commonly used for EV isolation than the above-mentioned methods, but it might soon flourish, as it can yield intact and contaminant-free EVs [16,23,24,25]. Moreover, SEC is not limited by any specific surface markers, unlike immuno-based isolations, which rely on using specific antibodies and result in isolation of only a subpopulation of EVs positive for the targeted marker. In SEC, particles are separated based on their size while they are carried by a fluid phase through a stationary phase. The stationary phase can be made of various materials, such as agarose or dextran, which produce a matrix of different pores and provide particle separation that is specifically efficient in the specified size ranges. However, these materials were originally designed for protein separation, and their suitability in the context of EV isolation is just starting to get unravelled. The first stationary phase successfully used for EV isolation by SEC was Sepharose CL-2B, applied for the separation of human plasma [26]. Soon followed EV isolations from human serum and plasma by Sepharose CL-6B and Sephacryl S-400 [27,28,29,30]. These materials are applied in SEC either as in-house designed columns with gravity flow or pre-packed columns for use with a pressure chromatography system. A much simpler option is now also commercially accessible: ready-to-use gravity-flow columns, which have already been successfully applied to isolate EVs from plasma and CSF [31]. Hence, SEC isolation of EVs comprises a wide range of different solutions, including the selection of stationary phase and column design. Different SEC designs might affect the efficiency of EV isolation, which can be crucial for clinical samples with a lower number of EVs, such as the cerebrospinal fluid (CSF) [31].

Being in direct contact with the central nervous system (CNS), CSF is a particularly valuable source of biomarkers for the ongoing processes in the nervous tissue [32,33]. CSF surrounds the CNS and supports its functioning by both physical and biochemical effects [34]. The physical effect of the CSF is to buffer the contact between the CNS and the surrounding bones, i.e., the scull protecting the brain and the spine around the spinal cord. Biochemically, CSF provides nourishment, waste disposal and intercellular communication [35,36]. There is growing evidence that some of these biochemical roles might be mediated by EVs, which puts EVs from CSF in the spotlight as carriers of biomarkers [37,38]. Indeed, EVs have been visualised in intracranial CSF from severe traumatic brain injury (TBI) patients and in lumbar CSF from control patients, and several studies describe the proteome and nucleic acid content of EVs from CSF of patients with Alzheimer’s disease, multiple sclerosis, and other CNS disorders [32,33,39,40,41,42,43]. Nevertheless, research of EVs from CSF is still affected by all of the previously described challenges of EV isolation. Furthermore, it is additionally burdened by two more factors: (1) CSF is accessed by invasive procedures, either by ventriculostomy when intracranial pressure needs to be monitored or by lumbar puncture for diagnostic purposes, which results in limited access to clinical CSF samples and a small volume of sample in the case of lumbar puncture; (2) CSF contains many fewer EVs than blood-derived samples, which poses higher requirements for EV isolation [31].

The objective of this study was to compare four gravity-based SEC methods in their ability to isolate EVs from intracranial CSF of patients with severe TBI. The SEC methods included three columns packed with Superose 6PG, Sephacryl S-400 or Sepharose CL-6B and the ready to use qEV10/70 nm column. The yield, purity and size distributions of the resulting isolated EVs were considered, as well as the simplicity and the duration of the separation process. Our results indicate that Sepharose CL-6B outperforms the other SEC methods applied here.

## 2. Materials and Methods

### 2.1. Patients

The study enrolled 3 adult patients with severe traumatic brain injury (TBI) who received ventriculostomy as part of their therapeutic intervention for intracranial pressure (ICP) monitoring and management in the intensive care unit of the Clinical Hospital Centre Rijeka and Pula General Hospital in Croatia. The patients were included in the study after one of their family members signed an informed consent form previously approved by the Ethical Committee. Patients were eligible if they were 18 to 80 years of age and had no immunological, malignant or chronic inflammatory disease. The research was conducted in accordance with the Declaration of Helsinki and local laws and regulations.

### 2.2. Cerebrospinal Fluid Sampling and Storing

CSF samples were obtained from ICP catheters using sterile 10 mL syringes under aseptic conditions by an appropriately trained physician. After being collected every 24 h during the first 3 days post injury, the samples were aliquoted into low-protein-binding tubes (Eppendorf, Germany) and stored at −80 °C. Sampling was conducted during 2020 and 2021. Samples had a maximum of two freezing–thawing cycles.

### 2.3. Size Exclusion Chromatography

Sepharose CL-6B (GE Healthcare, Uppsala, Sweden), Sephacryl S-400 HR (Cytiva, Uppsala, Sweden) and Superose 6 Prep Grade (Cytiva, Uppsala, Sweden) were packed into separate 1.5 × 50 cm glass columns fitted with a 30 µm bottom frit and equipped with flow adaptors (Bio-Rad Laboratories, Hercules, CA, USA). Approximately 75 mL of each resin was packed by applying distilled water under gravity flow. Columns were washed with at least 3 bed volumes of distilled water and equilibrated with 2 bed volumes of sterile PBS (Gibco, Grand Island, NY, USA) as running buffer prior to the first SEC run. Commercial qEV10/70 nm (Izon Science, Christchurch, New Zealand) column was washed and equilibrated in PBS as per the manufacturer’s instructions. The CSF-pool for SEC was created by combining equal volumes of 9 CSF samples from 3 severe TBI patients. The CSF-pool of 2.8 mL was loaded by a 5 mL sterile plastic syringe on each column and 50 fractions of 1.5 mL were collected in low-protein-binding tubes (Eppendorf, Hamburg, Germany) for each run. SEC was performed at room temperature and by gravity flow with the running buffer positioned 20 cm above the column. Columns were washed with at least two bed volumes of PBS between SEC runs.

### 2.4. Western Blot

Individual CSF samples, CSF-pool or SEC fractions were mixed with reducing 5× Laemmli buffer (1M Tris HCl pH 6.8, 50% glycerol (*v*/*v*), 10% SDS (*w*/*v*), 0.05% bromophenol blue (*w*/*v*), 2-mercaptoethanol) and boiled at 95 °C for 10 min. 25 µL of individual CSF and CSF-pool sample or 200 µL of SEC fractions was loaded onto 10% or 12% polyacrylamide gel and electrophoresed (Bio-Rad, Hercules, CA, USA) in 1× running buffer (25 mM Tris, 192 mM glycine and 0.1% SDS, pH 8.3) on 90 V to 150 V. Proteins were transferred to a 0.2 µm nitrocellulose membrane (GE Healthcare Life Science, Uppsala, Sweden) at the constant voltage of 20 V for 35 min using a semi-dry transfer unit (Hoefer, San Francisco, CA, USA). Membranes were stained with Ponceau S (0.1% Ponceau S in 5% acetic acid), followed by blocking with 5% milk in Tris-buffer saline (TBS, 20 mM Tris and 150 mM NaCl) for 15 min, and probed overnight at 4 °C in 5% bovine serum albumin (Roche Diagnostics, Mannheim, Germany)/TBS-T (TBS supplemented with 0.1% Tween 20) with rabbit monoclonal antibodies against CD81 (#56039), CD9 (#13174), apolipoprotein E (#13366) or albumin (#4929), and mouse monoclonal antibody against apolipoprotein AI (#3350), diluted 1:1000 (all antibodies purchased from Cell Signaling Technology, Danvers, MA, USA). Membranes were washed three times for 5 min in TBS-T and incubated with horseradish peroxidase-linked anti-rabbit (#7074) or anti-mouse (#7076) secondary antibodies (Cell Signaling Technology, Danvers, MA, USA) at room temperature for 30 min. After additional washing in TBS-T, the signal was visualised using SignalFire Plus ECL Reagent or SignalFire Elite ECL Reagent (Cell Signaling Technology, Danvers, MA, USA) and imaged with an imager (Alliance 4.7, Uvitec, Cambridge, UK or Li-COR Biosciences, Lincoln, NE, USA).

### 2.5. Slot Blot

SEC fractions were mixed with 5× Laemmli buffer, as stated above, but without glycerol and boiled at 95 °C for 10 min. Nitrocellulose membrane (GE Healthcare Life Science, Uppsala, Sweden) was soaked in distilled water and placed in the slot blot apparatus (Hoefer Scientific Instruments, San Francisco, CA, USA). SEC fractions in volume of 200 µL were loaded per slot and applied to the membrane by a vacuum pump. Each slot was then rinsed three times with 1 mL of PBS (Gibco, Grand Island, NY, USA) before removing the membrane from the slot blot apparatus. Membranes were stained with Ponceau S, blocked, and blotted for CD81, CD9, ApoAI, ApoE, and albumin antibodies, as stated above. Imaging and quantification were performed using the imager and Image Studio Digits software (LI-COR Biosciences, Lincoln, NE, USA). Arbitrary units obtained by quantification of single membrane were normalised against the highest value and plotted as percentages.

### 2.6. Tunable Resistive Pulse Sensing

Tunable resistive pulse sensing (TRPS) was used to measure nanoparticle concentration and size distribution in SEC fractions. The measurements were conducted using a qNano Gold instrument, NP400 nanopores and Control Suite 3.4 software according to the manufacturer’s instructions (Izon Science, Christchurch, New Zealand). Briefly, nanopores were wetted, coated, and equilibrated using the Izon reagent kit and calibration runs were performed with 350 nm carboxylated polystyrene beads at two pressure conditions: 6 mbar and 3 mbar. To ensure comparability between measurements, nanopore stretch and voltage were individually adjusted for each measurement session to detect particles in the 185–740 nm diameter range and maintain baseline current at approximately 135 nA. All samples were suspended at a 1:1 ratio in electrolyte solution provided in the Izon reagent kit and measured for 5 min at 6 mbar. If more than 500 particles were recorded in 5 min, an additional measurement of another 5 min was conducted at 3 mbar. To avoid cross-contamination, nanopore was flushed with electrolyte solution for at least 5 min at 20 mbar pressure between each sample measurement. The identical concentration fraction, i.e., the particle size range over which the particle concentration is calculated, from 185 to 740 nm, was applied for all measurements prior to any data analysis. Particle concentration and size distribution were determined only for TRPS-positive fractions whose measurements attained at least 500 nanoparticles at both pressure conditions after 5 min of recording. Recordings containing less than 500 nanoparticles in 5 min were categorised as negative, and corresponding fractions were considered as TRPS-negative fractions.

### 2.7. Zeta Potential Measurement

The zeta potential of nanoparticles was determined in SEC fractions by applying TRPS measurement on a qNano Gold instrument. Only fractions with at least 500 nanoparticles detected under two pressures as described above have been used for zeta potential measurement. Measurements were performed on NP400 nanopores. Initial calibration with carboxylated polystyrene beads was conducted at 1 pressure condition and 3 applied voltages, generating a corresponding baseline current of 85 nA, 110 nA, and 135 nA, followed by an additional measurement at a second pressure condition at the highest voltage applied in the previous step. Sample measurements were completed at the highest calibration voltage and pressure. At least 500 particles were recorded per each measurement.

### 2.8. Transmission Electron Microscopy

The samples were suspended and fixed in an equal volume of 6% paraformaldehyde (PFA)/3% glutaraldehyde (GA) solution for 15 min at room temperature, resulting in the fixed sample in 3% PFA/1.5%GA. A 10 μL drop of fixed sample was then deposited on a formvar/carbon-coated, 200-mesh copper grid (Ted Pella, Redding, CA, USA) that was placed on parafilm in a Petri dish for 60 min incubation on ice in a humidified atmosphere. After incubation, the excess liquid was removed by filter paper. The grids were incubated with 0.5% aqueous solution of uranyl acetate for a few seconds to contrast the sample. Then, the grids were consecutively transferred three times to 200 μL drops of distilled water and air dried. After drying, grids were observed with the 120-kV transmission electron microscope (JEM-2100 EXII, JEOL, Inc., Tokyo, Japan).

### 2.9. Statistics

Statistical analysis was performed using GraphPad Prism 8 (version 8.0.1, GraphPad software Inc., San Diego, CA, USA). The Shapiro–Wilk test was performed to assess the normality of data distributions, followed by one-way ANOVA with a post-hoc Tukey test. The statistical difference was accepted when *p* < 0.05.

## 3. Results

### 3.1. Severe Traumatic Brain Injury Patients

To compare EV isolation by different SEC methods, we applied CSF samples from severe TBI patients. Our previous data showed that intracranial CSF collected during the first days after the injury contained EVs [18,39]. Thus, we decided to create a CSF-pool by combining nine CSF samples obtained during the three days after the injury. Three patients with severe TBI were included (Table 1). The patients involved two males and one female with a mean age of 45 ± 3.6 years, Glasgow coma scale (GCS) score of 3–5 at admission and Glasgow outcome scale (GOS) score of 4 three months after discharge.

### 3.2. Intracranial Cerebrospinal Fluid during Three Days after Traumatic Brain Injury Contains Extracellular Vesicles and Lipoproteins

**Figure 1:**
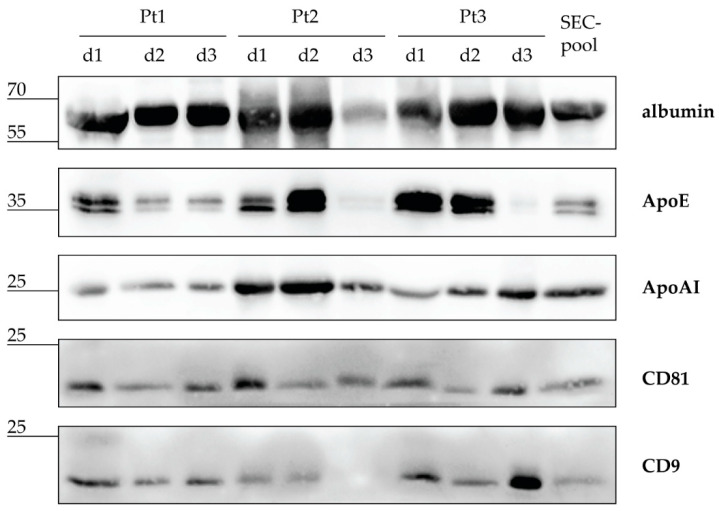
Intracranial cerebrospinal fluid (CSF) after severe traumatic brain injury contains EVs, lipoproteins, and albumin. CSF samples from three patients (Pt) collected during the first three days (d) after severe traumatic brain injury were pooled in equal volumes to make a CSF sample (CSF-pool) for size exclusion chromatography (SEC). Individual CSF samples and the CSF-pool were separated by electrophoresis and analysed by immunoblot with antibodies against albumin, apolipoprotein (Apo) E and AI, and EV proteins CD81 and CD9. Sizes of detected proteins are indicated in kilodaltons.

Both individual CSF samples and the resulting CSF-pool were screened for the presence of EVs, lipoproteins, and albumin by western blot analyses (Figure 1). The most abundantly detected protein in individual CSF samples was albumin, followed by lipoprotein markers ApoE and ApoAI, while EV markers CD81 and CD9 were detected at the lowest level. When patients were compared by protein kinetics, albumin was present at high levels all three days in all three patients, with only Patient 2 (Pt2) having a lower amount of albumin on day 3 (d3). Contrary to albumin, ApoE kinetics displayed a wider range in amounts between patients. Pt1 showed a moderate level of ApoE on day 1 and a lower level on days 2 and 3. However, Pt2 and Pt3 displayed moderate to high levels of ApoAI on d1 and d2, but a hardly detectable level on d3. ApoAI has been detected at low to moderate levels in all nine CSF samples. EV markers CD81 and CD9 showed mostly comparable levels among all 3 patients during the monitored 3 days, with the only exception of d3 for Pt2, when CD9 was not detected. Finally, the CSF-pool comprising all nine CSF samples contained the analysed proteins at detectable levels, indicating that the starting CSF-pool for SEC included free proteins, lipoproteins, and EVs.

### 3.3. Sepharose CL-6B, Superose 6PG, Sephacryl S-400 and qEV10/70 nm Column Differ in Technical Performance

After showing that the CSF-pool contains albumin, EVs, and lipoproteins, we proceeded with the SEC separation by applying gravity flow through the following SEC stationary phases, respectively: Superose 6PG, Sephacryl S-400, and Sepharose CL-6B, which have been packed into columns, and the commercially available ready-to-use column qEV10/70 nm (Table 2). To enable a direct comparison, each SEC method was conducted by loading the same volume of the identical CSF-pool and applying the same mobile phase. The fastest flow rate of 3.6 mL/min was measured for qEV10/70 nm, while Superose 6PG and Sephacryl S-400 had comparable rates of 0.2 mL/min and 0.3 mL/min, respectively. Sepharose CL-6B had the highest flow rate of 0.6 mL/min. Despite the differences in the flow rates, all four SEC methods required a similar mobile phase volume to elute nanoparticles (26–28 mL) and total proteins (68 to >74 mL).

### 3.4. Sepharose CL-6B Provides the Most Effective Isolation of Nanoparticles

To estimate the efficacy of SEC methods in separating CSF to nanoparticles and free proteins, 46 successive fractions were collected for each SEC method and individually analysed. First, a slot blot was performed followed by Ponceau staining to detect protein- enriched fractions (Figure S1a(A–D)). Sepharose CL-6B and Superose 6PG resulted in Ponceau positive fractions ranging from 24 to 43 and 23 to 45, respectively, thereby providing a collection of all protein-enriched fractions. On the other hand, Sephacryl S-400 and qEV10/70 nm resulted in a delayed range of Ponceau-positive fractions starting at fraction 27 and 28, respectively, and extending beyond the last collected fraction, 46. Thus, Sephacryl S-400 and qEV10/70 nm separated proteins into a larger number of later fractions containing lower protein concentrations.

TRPS measurements were performed only in approximately the first half of fractions since the protein-enriched fractions, as detected by Ponceau staining, caused clogging of the nanopore and hampered the measurement. With the predefined conditions for a fraction to reach either the required number of detected nanoparticles or the maximal time of measurement, we were able to distinguish between nanoparticle-positive and nanoparticle-negative fractions. The TRPS particle size distributions of the nanoparticle-positive fractions were continuous and unimodal, while the distributions of the nanoparticle-negative fractions were fragmented and multimodal, with insufficient number of particles for a reliable statistical analysis (Figure S1b(A,B)).

**Figure 2:**
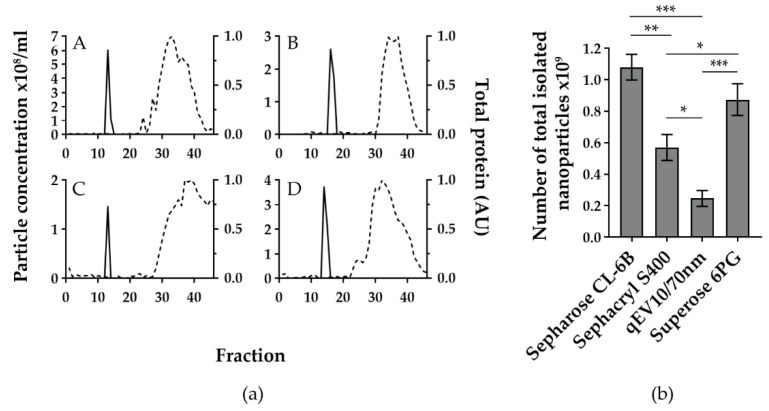
The four gravity-based size exclusion chromatographies (SEC) separate cerebrospinal fluid (CSF) to nanoparticle and free protein-enriched fractions but differ in yield of total nanoparticle number. (**a**) Sepharose CL-6B (**A**), Sephacryl S-400 (**B**), qEV10/70 nm (**C**), and Superose 6PG (**D**) were used to separate the same pool of intracranial CSF samples from severe traumatic brain injury patients. For each SEC method, 46 fractions of approximately 1.5 mL were collected and analysed for nanoparticle concentration (solid line), measured by tunable resistive pulse sensing (TRPS) and total protein content (dashed line) expressed as arbitrary units of quantified image of Ponceau-stained membrane after slot blot. Quantification was performed using Image J software. Shown are representative graphs out of three experiments for each SEC method. (**b**) Total number of isolated nanoparticles per analysed SEC method was calculated by adding up nanoparticle numbers from TRPS positive fractions. Mean values with standard deviations from three experiments are shown for each SEC method. * *p* < 0.01, ** *p* < 0.001, *** *p* < 0.0001 based on the one-way ANOVA with Tukey’s post-hoc test.

To compare the quality of SEC separations, TRPS measurements and quantified Ponceau staining of fractions were plotted together for each SEC method (Figure 2a(A–D)).

All SEC methods showed a narrow TRPS peak, mostly encompassing 2 fractions and positioned between fractions 12 and 17. The highest nanoparticle concentrations in TRPS positive fractions were 6.02 × 10^8^, 3.72 × 10^8^, 2.60 × 10^8^, and 1.46 × 10^8^ particles/mL for Sepharose CL-6B, Superose 6PG, Sephacryl S-400, and qEV10/70 nm, respectively.

When comparing the distance between the nanoparticle and the protein-enriched fractions, qEV10/70 nm and Sephacryl S-400 showed the largest distance between the TRPS peak and the Ponceau curve, followed by a somewhat shorter TRPS-Ponceau distance for Sephacryl S-400 and Sepharose CL-6B. However, when the total number of isolated nanoparticles (calculated as the sum of nanoparticle numbers from the nanoparticle enriched fractions) was compared, the differences between SEC methods became more obvious (Figure 2b). Sepharose CL-6B yielded the highest number, i.e., (1.08 ± 0.08) × 10^9^ nanoparticles.

### 3.5. Nanoparticles of Comparable Negative Charge Are Isolated in Similar Proportions by the Applied SEC Methods

**Figure 3:**
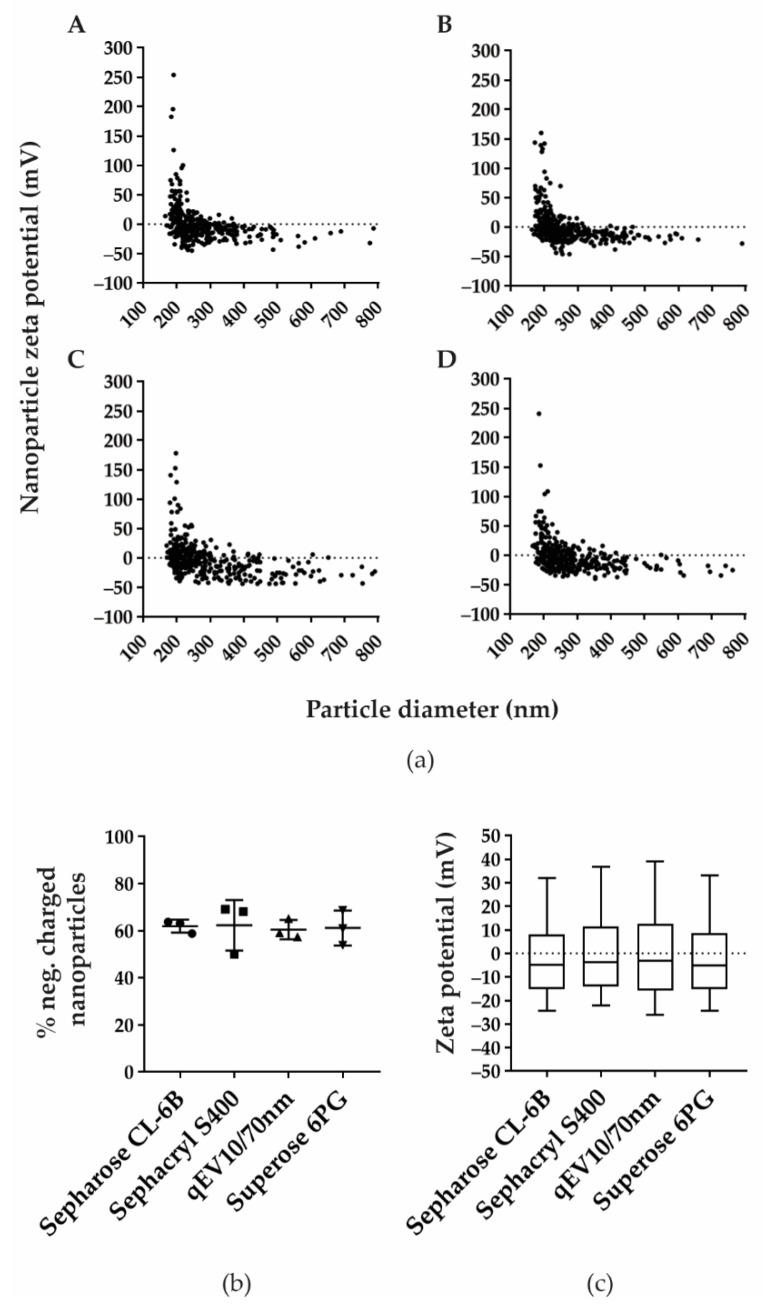
Zeta potential and percentage of negatively charged isolated nanoparticles are comparable in all four SEC methods. (**a**) Zeta potential was measured in TRPS positive fractions after separation by Sepharose CL-6B (**A**), Sephacryl S-400 (**B**), qEV10/70 nm (**C**), and Superose 6PG (**D**). Shown are representative distributions of nanoparticle zeta potential measured in millivolts (mV) and nanoparticle diameter measured in nanometres (nm). (**b**) Percentage of negatively charged nanoparticles are shown as mean values with their standard deviation from three experiments for each SEC method. (**c**) Measured zeta potentials are presented as median values and interquartile ranges (boxes) coupled with 10–90 percentile ranges (whiskers).

Since applied SEC methods showed a different efficacy in separation of nanoparticles from free proteins contained in CSF, we decided to further characterise nanoparticle-positive fractions by measuring zeta potential (Figure 3). The distribution of nanoparticle diameter and zeta potential was similar among the four SEC methods and resulted in 61.96%, 62.39%, 60.56% and 61.18% of negatively charged nanoparticles with zeta potential median −4.67 mV (−15.00; 8.17), −3.67 mV (−14.00; 11.33), and −3.00 mV (−15.67; 12.67), and −5.00 mV (−15.25; 8.67) for Sepharose CL-6B, Sephacryl S400, qEV10/70 nm, and Superose 6PG, respectively.

### 3.6. Sepharose CL-6B Separates EVs from Lipoproteins and Free Proteins

After discovering that Sepharose CL-6B effectively separated nanoparticles from the majority of proteins in human CSF, our next goal was to determine the identity of the isolated nanoparticles. Apparent candidates were EVs and lipoproteins, since both types of nanoparticles were detected in the CSF-pool applied for SEC separation (Figure 1). Moreover, EVs and lipoproteins overlap in size, and therefore they might co-elute in SEC-fractions and would not be distinguished by tunable pulse resistive sensing measurement [1,44]. Thus, we performed immunodetection of the corresponding protein markers in individual fractions collected after SEC with Sepharose CL-6B (Figure S2). The EV markers CD81 and CD9 were clearly detected on slot blot in a continuous range of fractions 13–16 and 13–18, respectively. Sporadic and weak CD81 signals were also found among later fractions in the 23–31 range. Albumin and the lipoprotein markers ApoAI and ApoE were noticed in a continuous range of fractions 27–37, 25–30, and 24–31, respectively. Albumin was also detected in fractions 39 and 40.

**Figure 4:**
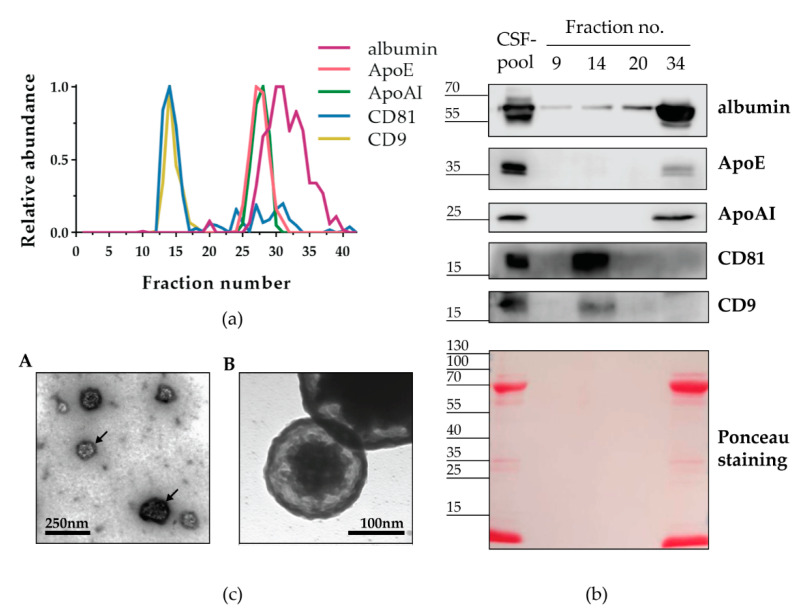
Nanoparticles isolated by Sepharose CL-6B contain CD9+ and CD81+ EVs. (**a**) CSF-pool from severe traumatic brain injury patients was separated by a gravity-flow column packed with sepharose CL-6B. Consecutively collected fractions indicated by numbers together with CSF-pool as positive control (PC) and PBS (mobile phase in SEC) as negative control (NC) were applied to nitrocellulose membrane by slot blot and analysed by immunodetection of indicated proteins. Shown are representative immunoblots after chemiluminescence. The chemiluminescence signal after immunodetection on slot blots for indicated proteins was quantified and normalised to the PC signal. Shown are relative protein abundances obtained by the quantification of representative immunoblots (**b**). Selected fractions were analysed by western blot after protein separation by SDS-PAGE. (**c**) Transmission electron microscopy micrograph showing round shaped extracellular vesicles measuring 100–130 nm in diameter and indicated by arrows (**A**). Higher magnification of electron micrograph showing extracellular vesicle with double-layer membrane measuring 175 nm (**B**).

To provide a better overview of the protein levels in individual fractions obtained with Sepharose CL-6B, we quantified the signals immunodetected on slot blot and plotted them on one graph (Figure 4a). All continuous ranges of positive fractions formed curves, which were narrow and symmetrical in the cases of CD81, CD9, ApoAI, and ApoE, and wider and less smooth in the case of albumin. Moreover, complete overlapping was observed for CD81 and CD9 curves as well as for ApoAI and ApoE curves. The albumin curve overlapped with the second part of both the ApoAI and ApoE curves. To confirm the specificity of immunodetection after slot blot of SEC-fractions, we also studied proteins of selected fractions by electrophoresis, followed by western blot analyses (Figure 4b). We chose fractions 9, 14, and 20 to represent SEC before, amid, and after nanoparticle-positive peak and fraction 34 to represent the free-protein peak (Figure 4a). After Ponceau staining, we observed no signal in fractions no. 9, 14, and 20, but we detected abundant proteins of roughly 70 kDa and less than 15 kDa in size, as well as low levels of proteins around 35 kDa and 100 kDa, in both fraction no. 34 and the CSF-pool used for the SEC separation. In the western blot analyses of the same fractions, we noticed signals of the expected size for every analysed protein. We detected CD81 and CD9 in fraction 14, but not in fraction 34, whereas the opposite was true for ApoAI and ApoE: we observed them in fraction 34, but not in fraction 14. Interestingly, the ratio of the CD81 and CD9 signals in the nanoparticle-enriched fractions differed from the ratio in the initial CSF-pool. To further confirm the presence of EVs in fraction 14, we performed electron microscopy analyses (Figure 4c). We detected round shaped extracellular vesicles measuring 100–130 nm in diameter.

## 4. Discussion

Extracellular vesicles (EVs) from cerebrospinal fluid (CSF) are expected to contain molecular cargo that could reveal biochemical changes in nervous tissue. To enable the detection of such biomarkers, it is important to extract EVs from clinical CSF in the highest possible purity, quantity, and heterogeneity. These criteria for EV-isolation might be met by size exclusion chromatography (SEC)—a well-established method for protein purification but not yet a common and standardised approach for EV-isolation. Here we demonstrated the differences between four SEC methods in their ability to enrich EVs from human CSF.

For this purpose, we analysed CSF samples obtained from adult severe traumatic brain injury (TBI) patients whose treatment included monitoring and management of intracranial pressure. Our study included intracranial CSFs drained during the first 3 days after injury. In the majority of individual CSFs, we detected high levels of albumin and apolipoproteins indicating blood content. This is consistent with the brain injury severity and the invasive procedure required for accessing the intracranial CSF [45]. EV protein markers CD81 and CD9 were found in all but one of the individual CSFs, confirming the finding of previous studies that these tetraspanins are commonly present in clinical CSF [39,46,47]. Nevertheless, CD81 and CD9 are ubiquitously expressed proteins and it is very likely also that at least some of CD81+ and CD9+ EVs derived from the blood [48,49]. While our immunoblot analyses of individual samples showed variable and not mutually consistent amounts of albumin and protein markers for EVs and lipoproteins, the pool created from nine post-TBI CSFs resulted in moderate levels of all detected proteins (Figure 1). The composition of such a CSF-pool is likely to be closer to that of average CSFs available for diagnostics and prognostics.

We applied the same volume of CSF-pool for four SEC columns based on different stationary phases: Sepharose CL-6B, Sephacryl S400, Superose 6PG and qEV10/70 nm. By this direct comparison we could show that all four analysed SEC methods were successful in separating nanoparticles of roughly 200 nm in size from free proteins, such as albumin. The importance of clear separation of EVs and free proteins has been recently demonstrated for the case of L1CAM, a transmembrane protein expressed on neural cells, which was thought to be suitable for immunoisolation of neuron-derived EVs [50]. However, L1CAM was proven to be present in free-protein fractions after Sepharose CL-6B chromatography and density gradient separation of CSF and plasma [51]. The same study also corroborates our findings that the SEC method is suitable for separation of EVs and lipoproteins (Figure 2). Lipoproteins overlap in size with EVs but contain a hydrophobic core surrounded by a lipid monolayer. They transport endogenous lipids and are abundant in blood and CSF [44]. As such, they can easily co-isolate with EVs and their characterisation is recommended to demonstrate the quality of EV isolates obtained from corresponding body fluids [5]. We show that Sepharose CL-6B is able to enrich lipoproteins in later fractions together with free proteins and is therefore suitable for reducing these contaminants in EV isolates.

Our results further indicate that the SEC stationary phase can largely influence the quantity of isolated EVs. Sepharose CL-6B and Superose 6PG provided significantly higher levels of concentrated EVs in comparison to Sephacryl S400 and qEV10/70 nm, which resulted in up to two to four times lower amounts of enriched EVs, respectively (Figure 2b). Moreover, Sepharose CL-6B provided a three times higher flow rate than Superose 6PG (Table 2). On the other hand, the simple usage combined with much faster flow rate work in favour of qEV10/70 nm (Table 2). In other studies of EV isolation, qEV10/70 nm has often been used to represent the SEC method and was shown to underperform in comparison with precipitation, membrane affinity, and density gradient methods [52]. Our data suggest that SEC efficiency largely depends on the stationary phase and that more details need to be considered when a SEC method is evaluated. Although we could show the separation of EVs from both the albumin as a prototype of free proteins and lipoproteins represented by ApoAI and ApoE, the purity of the EV-enriched fractions should be taken with caution. As demonstrated in Figure 4, albumin could be detected in the EV-enriched fractions even with western blot as a low-sensitivity method. However, it remains to be elucidated whether this albumin is associated with EVs or it represents contamination by free proteins. Another limitation of this study is that SEC might not result in the enrichment of all EVs and a portion of EVs might not be detected. Namely, we found significant differences in the quantity of EVs isolated by different SEC methods. This indicates that the stationary phase might interact with EVs in undesirable ways, including: (1) binding the EVs and thus preventing them from migrating with the mobile phase, and/or (2) influencing the migration of EVs in such a manner that they get spread in the large volume of the mobile phase and eluted in a greater number of fractions, resulting in diluted concentrations. These factors should be taken into account for further improvement of EV isolation by SEC. Additionally, other EV markers like Alix, Tsg101 and Rab proteins should be analysed to better understand which EVs are affected by this possible interaction with the stationary phase.

In conclusion, this study provides a protocol for gravity-based SEC that can be easily applied in any lab. We demonstrate that Sepharose CL-6B and Superose 6PG outperform Sephacryl S400 and qEV10/70 nm in enrichment of EVs from clinical CSF. The distribution of EVs in fractions is most likely affected by manual collection of fractions, and here applied gravity-based columns are not suitable for upgrading to large-scale analyses. Still, all compared SEC stationary phases were able to separate EVs from lipoproteins and free proteins and further improvements might be achieved by choosing and/or further developing stationary phase materials.

## Figures and Tables

**Table 1:** Description of severe traumatic brain injury patients included in the study.

Patient	Age	Gender	Mechanism of Injury	GCS ^1^ at Admission	GCS ^1^ at Discharge	GOS ^2^ Three Months after Discharge	Intracranial Pathology
1	44	M	Fall from height	3	14	4	Epidural haematoma
2	49	F	Motor vehicle accident	5	14	4	Intracerebral haematoma, traumatic subarachnoid haemorrhage
3	42	M	Motor vehicle accident	3	14	4	Traumatic subarachnoid haemorrhage, concussion foci, frontal, temporal, occipital

^1^ Glasgow Coma Scale; ^2^ Glasgow Outcome Scale.

**Table 2:** Design and performance of four size exclusion chromatography (SEC) methods based on gravity flow and applied to separate cerebrospinal fluid from patients with severe traumatic brain injury.

Gravity Flow SEC	Column Packing Required	Average Flow Rate in mL/min	Volume of Mobile Phase Required for Elution of	
			Nanoparticles	Total Proteins
Superose 6PG	yes	0.2	26 mL	68 mL
Sephacryl S-400	yes	0.3	28 mL	68 mL
Sepharose CL-6B	yes	0.6	25 mL	70 mL
qEV10/70 nm	no	3.6	26 mL	>74 mL

## Supplementary Materials

Shown are Ponceau stained membranes with fractions obtained from the analysed SEC methods and loaded to the membrane by the slot blot technique. The particle size distributions gained by the TRPS measurement are presented for two SEC-fractions demonstrating the difference between a nanoparticle-positive and a nanoparticle negative SEC-fraction. Immunodetections are depicted for slot blots with fractions collected after separation by Sepharose CL-6B. The following supporting information can be downloaded at: https://www.mdpi.com/article/10.3390/biomedicines10040785/s1, Figure S1: Detection of nanoparticle and free protein levels in fractions after SEC. Figure S2: Immunodetection of EV, lipoprotein and free protein markers in individual SEC-fractions after separation by Sepharose CL-6B.
