# Supplementary material for: Extracellular Vesicles from Human Cerebrospinal Fluid Are Effectively Separated by Sepharose CL-6B—Comparison of Four Gravity-Flow Size Exclusion Chromatography Methods
Source: Biomedicines. 2022 Mar 27;10(4):785. doi: 10.3390/biomedicines10040785 (PMC9032713; doi:10.3390/biomedicines10040785)
Supplement: Supplementary file 1 [file biomedicines-10-00785-s001.zip › biomedicines-1622661-supplementary.pdf]

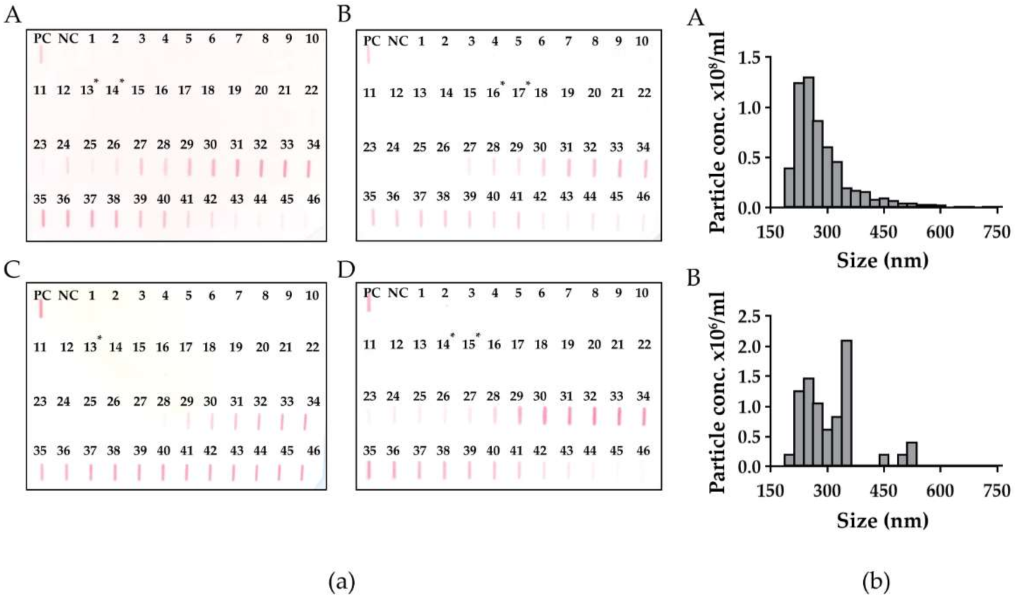

Figure S1. Detection of nanoparticle and free protein levels in fractions after SEC. (a) Fractions collected after cerebrospinal fluid (CSF) separation by Sepharose CL-6B (A), Sephacryl S-400 (B), qEV10/70nm (C) or Superose 6PG (D) were loaded in equal volume on nitrocellulose membrane followed by Ponceau staining. CSF-pool before SEC separation was used as positive control (PC) and SEC mobile phase was used as negative control (NC). Numbers indicate consecutively collected fractions. Shown are representative Ponceau images for each SEC method. Nanoparticle enriched fractions are denoted by asterisk. (b) Nanoparticle concentration was measured by tunable resistive pulse sensing (TRPS). A fraction was considered as TRPS positive (A) if minimally 500 nanoparticles were detected during the 5-minute measurement. Fractions with lower rate of nanoparticle detection by TRPS and thus with less than 500 nanoparticles detected, were categorised as TRPS negative (B). Shown are representative nanoparticle distributions for both TRPS positive and negative fractions.

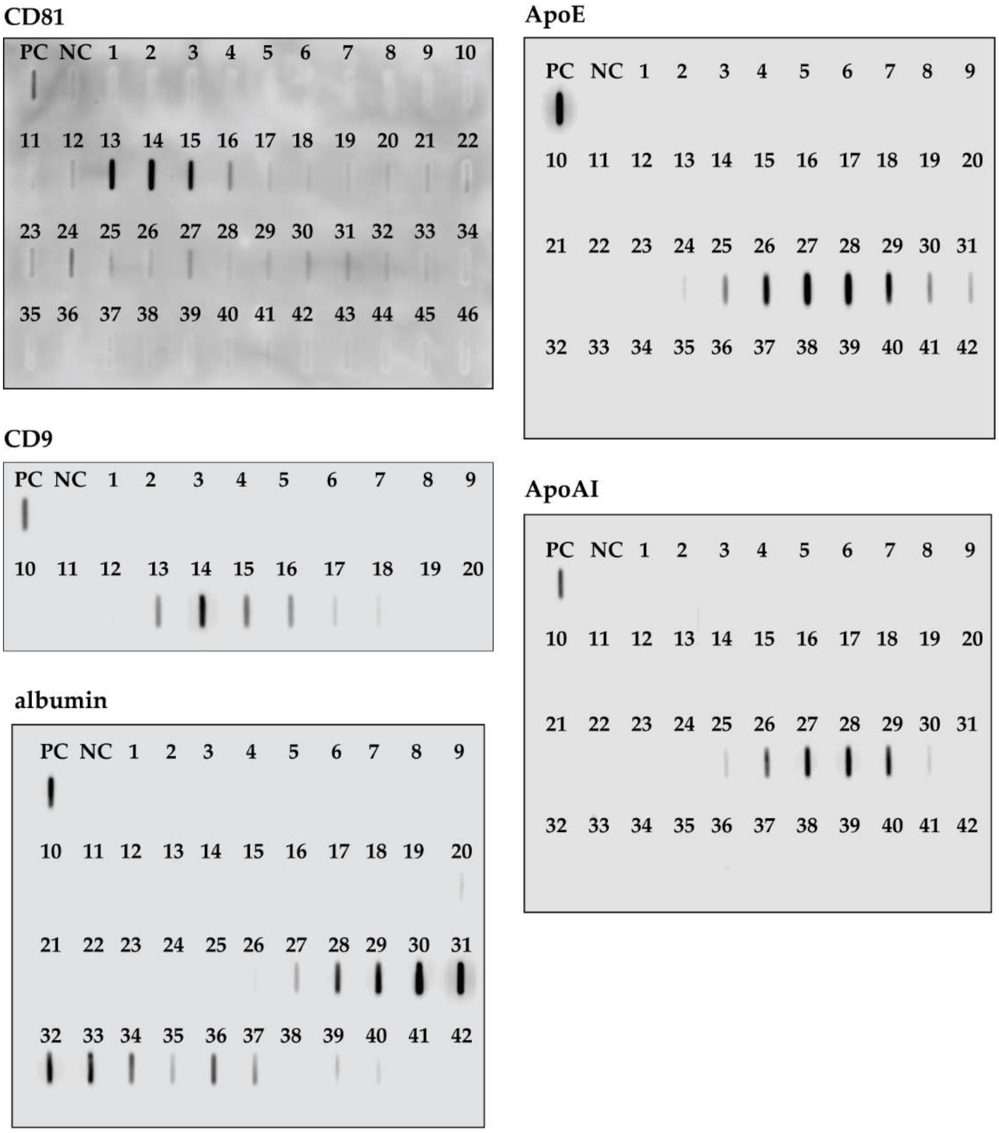

Figure S2. Immunodetection of EV, lipoprotein and free protein markers in individual SEC-fractions after separation by Sepharose CL-6B. Fractions collected after separating the CSF-pool by Sepharose CL-6B chromatography were loaded on nitrocellulose membrane using a slot blot apparatus. CSF-pool and SEC mobile phase were loaded as positive control (PC) and negative control (NC), respectively. Indicated proteins were immunodetected by corresponding antibodies. Collected fractions are indicated by numbers.
